# Evidence for similar structural brain anomalies in youth and adult attention-deficit/hyperactivity disorder: a machine learning analysis

**DOI:** 10.1038/s41398-021-01201-4

**Published:** 2021-02-01

**Authors:** Yanli Zhang-James, Emily C. Helminen, Jinru Liu, The ENIGMA-ADHD Working Group, Geraldo F. Busatto, Anna Calvo, Mara Cercignani, Tiffany M. Chaim-Avancini, Matt C. Gabel, Neil A. Harrison, Luisa Lazaro, Sara Lera-Miguel, Mario R. Louza, Rosa Nicolau, Pedro G. P. Rosa, Martin Schulte-Rutte, Marcus V. Zanetti, Sara Ambrosino, Philip Asherson, Tobias Banaschewski, Alexandr Baranov, Sarah Baumeister, Ramona Baur-Streubel, Mark A. Bellgrove, Joseph Biederman, Janita Bralten, Ivanei E. Bramati, Daniel Brandeis, Silvia Brem, Jan K. Buitelaar, Francisco. X. Castellanos, Kaylita C. Chantiluke, Anastasia Christakou, David Coghill, Annette Conzelmann, Ana I. Cubillo, Anders M. Dale, Patrick de Zeeuw, Alysa E. Doyle, Sarah Durston, Eric A. Earl, Jeffrey N. Epstein, Thomas Ethofer, Damien A. Fair, Andreas J. Fallgatter, Thomas Frodl, Tinatin Gogberashvili, Jan Haavik, Catharina A. Hartman, Dirk J. Heslenfeld, Pieter J. Hoekstra, Sarah Hohmann, Marie F. Høvik, Neda Jahanshad, Terry L. Jernigan, Bernd Kardatzki, Georgii Karkashadze, Clare Kelly, Gregor Kohls, Kerstin Konrad, Jonna Kuntsi, Klaus-Peter Lesch, Astri J. Lundervold, Charles B. Malpas, Paulo Mattos, Hazel McCarthy, Mitul A. Mehta, Leyla Namazova-Baranova, Joel T. Nigg, Stephanie E. Novotny, Ruth L. O’Gorman Tuura, Eileen Oberwelland Weiss, Jaap Oosterlaan, Bob Oranje, Yannis Paloyelis, Paul Pauli, Kerstin J. Plessen, J. Antoni Ramos-Quiroga, Andreas Reif, Liesbeth Reneman, Katya Rubia, Anouk Schrantee, Lena Schwarz, Lizanne J. S. Schweren, Jochen Seitz, Philip Shaw, Tim J. Silk, Norbert Skokauskas, Juan Carlos Soliva Vila, Michael C. Stevens, Gustavo Sudre, Leanne Tamm, Paul M. Thompson, Fernanda Tovar-Moll, Theo G. M. van Erp, Alasdair Vance, Oscar Vilarroya, Yolanda Vives-Gilabert, Georg G. von Polier, Susanne Walitza, Yuliya N. Yoncheva, Georg C. Ziegler, Barbara Franke, Martine Hoogman, Stephen V. Faraone

**Affiliations:** 1 Department of Psychiatry and Behavioral Sciences, SUNY Upstate Medical University, Syracuse, NY USA; 2 Department of Psychology, Syracuse University, Syracuse, NY USA; 3 University of Illinois at Urbana-Champaign, Champaign, IL USA; 4 Department of Human Genetics, Radboud University Medical Center, Nijmegen, The Netherlands; 5 Donders Institute for Brain, Cognition and Behaviour, Nijmegen, The Netherlands; 6 Department of Psychiatry, Radboud University Medical Center, Nijmegen, The Netherlands; 7 Department of Neuroscience and Physiology, SUNY Upstate Medical University, Syracuse, NY USA; 8 Laboratory of Psychiatric Neuroimaging (LIM21), Hospital das Clinicas HCFMUSP, Faculdade de Medicina, Universidade de Sao Paulo, SP, Brazil; 9 Department and Institute of Psychiatry, Faculty of Medicine, University of Sao Paulo, Sao Paulo, Brazil; 10 Magnetic Resonance Image Core Facility, Institut d’Investigacions Biomèdiques August Pi i Sunyer (IDIBAPS), Barcelona, Spain; 11 Department of Neuroscience, Brighton and Sussex Medical School, Falmer, Brighton UK; 12 Laboratory of Psychiatric Neuroimaging (LIM-21), Department and Institute of Psychiatry, Faculty of Medicine, University of São Paulo, Sao Paulo, Sao Paulo, Brazil; 13 Center for Interdisciplinary Research on Applied Neurosciences (NAPNA), University of São Paulo, Sao Paulo, Brazil; 14 Sussex Partnership NHS Foundation Trust, Swandean, East Sussex UK; 15 Institut d’Investigacions Biomèdiques August Pi i Sunyer (IDIBAPS), Barcelona, Spain; 16 Biomedical Network Research Center on Mental Health (CIBERSAM), Barcelona, Spain; 17 Department of Medicine, University of Barcelona, Barcelona, Spain; 18 Department of Child and Adolescent Psychiatry and Psychology, Institute of Neurosciencies, Hospital Clínic, Barcelona, Spain; 19 Department of Psychiatry, Faculty of Medicine, University of São Paulo, São Paulo, Brazil; 20 Child Neuropsychology Section, University Hospital Aachen, Aachen, Germany; 21 JARA Translational Brain Medicine, Research Center Juelich, Aachen, Germany; 22 NICHE Lab, Department of Psychiatry, University Medical Center Utrecht, Utrecht, The Netherlands; 23 Social, Genetic and Developmental Psychiatry Centre, Institute of Psychiatry, Psychology and Neuroscience, King’s College London, London, UK; 24 Department of Child and Adolescent Psychiatry and Psychotherapy, Central Institute of Mental Health, Medical Faculty Mannheim/Heidelberg University, Mannheim, Germany; 25 National Medical Research Center for Children’s Health, Moscow, Russia; 26 Department of Biological Psychology, Clinical Psychology, and Psychotherapy, University of Würzburg, Würzburg, Germany; 27 Turner Institute for Brain and Mental Health and School of Psychological Sciences, Monash University, Melbourne, Australia; 28 Clinical and Research Programs in Pediatric Psychopharmacology and Adult ADHD, Department of Psychiatry, Massachusetts General Hospital, Boston, MA USA; 29 Department of Psychiatry, Massachusetts General Hospital, Harvard Medical School, Boston, MA USA; 30 D’Or Institute for Research and Education, Rio de Janeiro, Brazil; 31 Department of Child and Adolescent Psychiatry and Psychotherapy, Psychiatric Hospital, University of Zurich, Zurich, Switzerland; 32 Neuroscience Center, University of Zurich and ETH Zurich, Zurich, Switzerland; 33 Karakter Child and Adolescent Psychiatry University Center, Nijmegen, The Netherlands; 34 Department of Child and Adolescent Psychiatry, NYU Langone Medical Center, New York, NY USA; 35 Nathan Kline Institute for Psychiatric Research, Orangeburg, NY USA; 36 Section of Biomedical Image Analysis, Department of Radiology, University of Pennsylvania, Philadelphia, PA USA; 37 Department of Child and Adolescent Psychiatry, Institute of Psychiatry, Psychology and Neuroscience, King’s College London, London, UK; 38 School of Psychology and Clinical Language Sciences, Centre for Integrative Neuroscience and Neurodynamics, University of Reading, Reading, UK; 39 Departments of Paediatrics and Psychiatry, The University of Melbourne, Melbourne, Australia; 40 Murdoch Children’s Research Institute, The University of Melbourne, Melbourne, Australia; 41 Division of Neuroscience, University of Dundee, Dundee, UK; 42 Department of Child and Adolescent Psychiatry, Psychosomatics and Psychotherapy, University Hospital of Tübingen, Tübingen, Germany; 43 Department of Psychology (Clinical Psychology II), PFH – Private University of Applied Sciences, Göttingen, Germany; 44 Departments of Neurosciences, Radiology, and Psychiatry, UC San Diego, San Diego, CA USA; 45 Center for Multimodal Imaging and Genetics (CMIG), UC San Diego, San Diego, CA USA; 46 Center for Genomic Medicine, Massachusetts General Hospital, Harvard Medical School, Boston, MA USA; 47 Department of Behavioral Neuroscience, Oregon Health & Science University, Portland, OR USA; 48 Division of Behavioral Medicine and Clinical Psychology, Cincinnati Children’s Hospital Medical Center, Cincinnati, OH USA; 49 Department of Pediatrics, University of Cincinnati College of Medicine, Cincinnati, OH USA; 50 Department of Psychiatry and Psychotherapy, University Hospital of Tübingen, Tübingen, Germany; 51 Department of Biomedical Magnetic Resonance, University of Tübingen, Tübingen, Germany; 52 Department of Psychiatry, Oregon Health & Science University, Portland, OR USA; 53 LEAD Graduate School, University of Tübingen, Tübingen, Germany; 54 Department of Psychiatry and Psychotherapy, Otto von Guericke University, Magdeburg, Germany; 55 Department of Psychiatry, Trinity College Dublin, The University of Dublin, Dublin, Ireland; 56 German Center for Neurodegenerative Diseases (DZNE), Magdeburg, Germany; 57 Laboratory of Neurology and Cognitive Health, National Medical Research Center for Children’s Health, Moscow, Russia; 58 K.G. Jebsen Centre for Neuropsychiatric Disorders, Department of Biomedicine, University of Bergen, Bergen, Norway; 59 Division of Psychiatry, Haukeland University Hospital, Bergen, Norway; 60 Department of Psychiatry, Interdisciplinary Center Psychopathology and Emotion Regulation (ICPE), University Medical Center Groningen, University of Groningen, Groningen, The Netherlands; 61 Faculty of Behavioural and Movement Sciences, Vrije Universiteit Amsterdam, Amsterdam, The Netherlands; 62 Department of Clinical Medicine, University of Bergen, Bergen, Norway; 63 Imaging Genetics Center, Stevens Institute for Neuroimaging & Informatics, Keck School of Medicine, University of Southern California, Los Angeles, CA USA; 64 Center for Human Development, UC San Diego, San Diego, CA USA; 65 School of Psychology and Department of Psychiatry at the School of Medicine, Trinity College Dublin, the University of Dublin, Dublin, Ireland; 66 Trinity College Institute of Neuroscience, Trinity College Dublin, The University of Dublin, Dublin, Ireland; 67 Child Neuropsychology Section, University Hospital RWTH Aachen, Aachen, Germany; 68 JARA Institute Molecular Neuroscience and Neuroimaging (INM-11), Institute for Neuroscience and Medicine, Research Center Jülich, Jülich, Germany; 69 Division of Molecular Psychiatry, Center of Mental Health, University of Würzburg, Würzburg, Germany; 70 Laboratory of Psychiatric Neurobiology, Institute of Molecular Medicine, I.M. Sechenov First Moscow State Medical University, Moscow, Russia; 71 Department of Neuroscience, School for Mental Health and Neuroscience (MHeNS), Maastricht University, Maastricht, The Netherlands; 72 Department of Biological and Medical Psychology, University of Bergen, Bergen, Norway; 73 Developmental Imaging Group, Murdoch Children’s Research Institute, The University of Melbourne, Melbourne, Australia; 74 Clinical Outcomes Research Unit (CORe), Department of Medicine, Royal Melbourne Hospital, The University of Melbourne, Melbourne, Australia; 75 Melbourne School of Psychological Sciences, The University of Melbourne, Melbourne, Australia; 76 Federal University of Rio de Janeiro, Rio de Janeiro, Brazil; 77 Centre of Advanced Medical Imaging, St James’s Hospital, Dublin, Ireland; 78 Department of Neuroimaging, Institute of Psychiatry, Psychology and Neuroscience, King’s College London, London, UK; 79 Olin Neuropsychiatry Research Center, Hartford Hospital, Hartford, CT USA; 80 Center for MR Research, University Children’s Hospital, Zurich, Switzerland; 81 Zurich Center for Integrative Human Physiology (ZIHP), Zürich, Switzerland; 82 Child and Adolescent Psychiatry, University Hospital RWTH Aachen, Aachen, Germany; 83 Cognitive Neuroscience (INM-3), Institute for Neuroscience and Medicine, Research Center Jülich, Jülich, Germany; 84 Clinical Neuropsychology Section, Vrije Universiteit Amsterdam, Amsterdam, The Netherlands; 85 Emma Children’s Hospital Amsterdam Medical Center, Amsterdam, The Netherlands; 86 Department of Pediatrics, VU Medical Center, Vrije Universiteit Amsterdam, Amsterdam, The Netherlands; 87 Child and Adolescent Mental Health Centre, Copenhagen, Denmark; 88 Division of Child and Adolescent Psychiatry, Department of Psychiatry, University Hospital Lausanne, Lausanne, Switzerland; 89 Department of Psychiatry, Hospital Universitari Vall d’Hebron, Barcelona, Catalonia Spain; 90 Group of Psychiatry, Addictions and Mental Health, Vall d’Hebron Research Institute, Barcelona, Barcelona Spain; 91 Biomedical Network Research Centre on Mental Health (CIBERSAM), Barcelona, Catalonia Spain; 92 Department of Psychiatry and Forensic Medicine, Universitat Autonoma de Barcelona, Barcelona, Spain; 93 Department of Psychiatry, Psychosomatic Medicine and Psychotherapy, University Hospital Frankfurt, Frankfurt, Germany; 94 Department of Radiology and Nuclear Medicine, Amsterdam University Medical Centers, Amsterdam, The Netherlands; 95 Brain Imaging Center, Amsterdam University Medical Centers, Amsterdam, The Netherlands; 96 National Human Genome Research Institute, Bethesda, MD USA; 97 National Institute of Mental Health, Bethesda, MD USA; 98 School of Psychology, Deakin University, Geelong, Australia; 99 Developmental Imaging Group, Murdoch Children’s Research Institute, The University of Melbourne, Melbourne, Australia; 100 Department of Paediatrics, The University of Melbourne, Melbourne, Australia; 101 Institute of Mental Health, Norwegian University of Science and Technology, Trondheim, Norway; 102 Department of Psychiatry, School of Medicine, Yale University, New Haven, CT USA; 103 Department of Pediatrics, Cincinnati Children’s Hospital Medical Center, Cincinnati, OH USA; 104 College of Medicine, University of Cincinnati, Cincinnati, OH USA; 105 Imaging Genetics Center, Stevens Institute for Neuroimaging & Informatics, Keck School of Medicine, University of Southern California, Los Angeles, CA USA; 106 Morphological Sciences Program, Federal University of Rio de Janeiro, Rio de Janeiro, Brazil; 107 Clinical and Translational Neuroscience Laboratory, Department of Psychiatry and Human Behavior, University of California Irvine, Irvine, CA USA; 108 Hospital del Mar Medical Research Institute (IMIM), Barcelona, Spain; 109 Instituto ITACA, Universitat Politècnica de València, València, Spain

**Keywords:** Predictive markers, Psychiatric disorders

## Abstract

Attention-deficit/hyperactivity disorder (ADHD) affects 5% of children world-wide. Of these, two-thirds continue to have impairing symptoms of ADHD into adulthood. Although a large literature implicates structural brain differences of the disorder, it is not clear if adults with ADHD have similar neuroanatomical differences as those seen in children with recent reports from the large ENIGMA-ADHD consortium finding structural differences for children but not for adults. This paper uses deep learning neural network classification models to determine if there are neuroanatomical changes in the brains of children with ADHD that are also observed for adult ADHD, and vice versa. We found that structural MRI data can significantly separate ADHD from control participants for both children and adults. Consistent with the prior reports from ENIGMA-ADHD, prediction performance and effect sizes were better for the child than the adult samples. The model trained on adult samples significantly predicted ADHD in the child sample, suggesting that our model learned anatomical features that are common to ADHD in childhood and adulthood. These results support the continuity of ADHD’s brain differences from childhood to adulthood. In addition, our work demonstrates a novel use of neural network classification models to test hypotheses about developmental continuity.

## Introduction

Attention-deficit/hyperactivity disorder (ADHD) is a common disorder affecting 5% of children and 3% of adults^1^. It is associated with injuries^2^, traffic accidents^3^, increased health care utilization^4,5^, substance abuse^6,7^, criminality^8^, unemployment^1^, divorce^9^, suicide^10,11^, AIDS risk behaviors^12^, and premature mortality^13^. The cost of adult ADHD to society is between $77.5 and $115.9 billion each year^14^.

ADHD is highly heritable (76% heritability)^15^. A role for brain dysfunction in the etiology of ADHD was suspected for some time by the mechanism of action of the medications that treat ADHD^16^, as well as supported by findings from genome-wide association studies (GWAS)^17,18^. Although many prior magnetic resonance imaging (MRI) studies had suggested structural and functional differences between the brains of children with ADHD and those without^19–25^, machine learning (ML) MRI diagnostic classifiers for ADHD have reported inconsistent results. We and others have examined this body of literature and reported large variations in choices of MRI modalities, ML models, cross-validation and testing methods, and sample sizes. Notably, many prior studies risked data leakage and accuracy inflation by using cross-validation methods without an independent test set^26^. In addition, the largest dataset that ML classifiers have used thus far was the ADHD-200 Global Competition dataset consisting of 776 children, adolescents, and young adults (7–21 years old^27^). Only a few studies examined classifiers for adults with ADHD and they all used extremely small datasets (<100 subjects^28–30^).

The Enhancing Neuro Imaging Genetics Through Meta-Analysis (ENIGMA) ADHD Working Group created a large collaborative dataset with sufficient power to detect small effects. The ENIGMA-ADHD working group found small, statistically significant sub-cortical volumetric reductions^31^, cortical thinning, and reduced surface area^32^ to be associated with ADHD in children but not adults. Two-thirds of youth with ADHD will continue to have impairing symptoms of the disorder into young adulthood and that persistence continues to decline with age^33^. The term adult ADHD refers to childhood onset ADHD that has persisted into adulthood, which is how it is defined in DSM 5 and in the ENIGMA-ADHD studies. The ENIGMA-ADHD study found small but significant ADHD vs. control differences in regional volumes and cortical thicknesses for children but not adolescents or adults^19,34,35^. Other studies show that ADHD participants whose brains become more neurotypical were more likely than others to show remission of symptoms^36,37^. But, although these longitudinal studies show reductions in case vs. control differences, they also suggest that those differences should be evident to some degree in cases that persist into adulthood.

Although the expectation of finding substantial continuity between childhood and adult ADHD has been widely accepted^33,38,39^ and recently confirmed by a large GWAS^40^, this idea has been challenged^41^. Thus, given these prior data and the controversy about the continuity of ADHD into adulthood, we sought to test the idea that the ADHD-associated volumetric reductions seen in children with ADHD would be detected in adults with ADHD by applying ML algorithms. Given that symptoms and impairments persist into adulthood for most children with ADHD^42,43^, we hypothesized that ADHD-related brain structure differences in adults would be consistent with those observed in children.

## Materials and methods

### MRI samples

The current study was approved by all contributing members of the ENIGMA-ADHD Working Group, which provided T1-weighted structural MRI (sMRI) data from 4183 subjects from 35 participating sites (by Aug. 2019). Each participating site had approval from its local ethics committee to perform the study and to share de-identified, anonymized individual data. Images were processed using the consortium’s standard segmentation algorithms in FreeSurfer (V5.1 and V5.3)^31^. A total of 151 variables were used including 34 cortical surface areas, 34 cortical thickness measurements, and 7 subcortical regions from each hemisphere, and intracranial volume (ICV). Subjects missing more than 50% of variables were removed. Remaining missing values and outliers (outside of 1.5 times the interquartile range (iqr 1.5)) were replaced with imputed values using multiple imputation with chained equations in STATA15. The final ML dataset consisted 4042 subjects from 35 sites, among which 45.8% were non-ADHD controls (*n* = 1850, male to female ratio (m/f) = 1.42) and 54.2% ADHD participants (*n* = 2192, m/f = 2.79). Ages ranged from four to 63 years old; 60.7% were children (age <18 years, *n* = 2454) and 39.3% were adults (age ≥18 years, *n* = 1588). ADHD diagnosis was significantly biased by sex (X^2^_(1)_ = 66.9, *p* < 0.0001), sites (X^2^_(1)_ = 146.73, *p* < 0.0001), and age (X^2^_(1)_ = 4.28, *p* = 0.04).

To balance the confounding factors, we took the following steps. First, we randomly assigned samples to training (~70%), validation (~15%), and test (~15%) subsets within each diagnosis, sex, age subgroup (child vs. adult), and site to ensure that the train/validation/test subsets have the same composition of these variables. Twelve sites that provided only cases or only controls (total 203 subjects) were excluded during the initial train/validation/test split because their samples cannot provide an unbiased learning during the training and validation steps. These samples were added to the test set for final test evaluation. Supplementary Table 1 shows the sample splitting from each site. Next, we balanced the training set for the case and control groups within each sex, age, and site subgroup by random oversampling of the under-represented diagnostic group, a procedure commonly used to deal with class imbalance. The resulting balanced training set is described in Table 1. The validation and test sets were not balanced by age, sex, and site, however due to our sample splitting procedures, they contain the same demographic samples as the training set. In addition, the test set also contains samples from sites that had been excluded from the training set due to not having a site-specific control group.

**Table 1:** Training set sample characteristics after balancing for age and sex.

Diagnosis		Child (age <18)		Adult (age ≥18)	
		Female	Male	Female	Male
Control	N of subjects	352	714	224	373
	Mean age	11.3	11.6	31.9	28.1
	SD of age	2.9	2.9	11.5	9.4
ADHD	N of subjects	352	714	224	373
	Mean age	11.0	11.8	32.2	28.8
	SD of age	2.6	2.7	10.6	9.4

*SD* standard deviation, *N* total numbers.

### Feature preprocessing

The high correlation among the 151 MRI features suggested the need for feature dimension reduction. Many prior studies have opted for feature selection in which the most important features were retained rather than using all MRI features. Although this approach reduces the numbers of input features, it does not remove the highly correlated relationships among the selected features. As prior MRI studies also suggested small but widespread differences between children with and without ADHD, we chose to use principal factors factor analysis (PFFA) for dimension reduction. With varimax rotation, PFFA on sMRI features of the training set identified 46 factors that explained >90% of the variance. This means that the reduced numbers of 46 non-correlated factors were able to represent majority (>90%) of the variance within the training dataset. We then computed factor scores for subjects in the validation and test sets based on the training set PFFA. We compared the original MRI and PFFA features in a screening pipeline for nine different ML models (see below) to determine which set of features were better for the classifiers.

### Machine learning framework

Our ML framework starts with a screening pipeline in which nine different ML models were thoroughly evaluated. We used only training and validation sets for this purpose and we also compared the results of the original MRI features and the PFFA factors. Children and adults were combined for the screening analysis. The screening pipeline utilized Scikit‐Learn’s grid search algorithm^44^ to search a large hyperparameter space for each of the models (see Supplementary Fig. 1 for details on these models and their hyperparameter spaces). We then compared both the training and validation scores of all the possible combinations of the hyperparameter sets. We used the area under the receiver operating characteristic (ROC) curves (AUC) as a measure of accuracy. To avoid overfitting, we chose the model having the highest validation AUC and smaller training AUC. Because multilayer perceptron (MLP) neural network models were found to be better than other models in meeting this criterion, we used MLP in the following analysis.

More detailed hyperparameter tuning for MLP was carried out using the Keras API (version 2.3.1), the TensorFlow library (version 1.14.0), and HyperOpt^45^. The neural network hyperparameters and their spaces are: the numbers of layers (1–3, model deteriorates quickly when more than 3 layers were used), numbers of units in each layer (4–500) and dropout rates in each layer (0.1–0.9), learning rate (0.00001–0.01) and batch normalization size (4–256). These hyperparameters were chosen for the HyperOpt tuning because of their important role in effective learning, avoiding local minimum and overfitting. The numbers of layers and units determines the complexity of the model. The ideal complexity of the neural network ensures a converging model that was able to learn the predictive features but not overfit the training examples. Early stopping was also implemented to avoid overfitting. We tested different activation functions (relu, selu, tanh), and optimizers (Adam, SGD, RMSprop, Adagrad, Adamax, Nadam). We used binary cross entropy as the loss function. Best model architecture and hyperparameters were chosen based on the lowest total validation loss. Final test scores were obtained on the test set with ensemble learning approach^46^. All ML algorithms were written in Python 3.5.

### Analysis pipelin**e**

Our main analysis pipeline starts with two base models that used data from the corresponding age groups during the model training and validation phase and tested also on data from their corresponding age groups. The child model used only child samples during model training, validation, and hyperparameter optimization, and tested on child test set. The adult model, similarly, was trained and validated on the adult samples and tested on the adult test set. We examined models using MRI features only, as well as those included age and sex information. We also trained a combined model that uses all the training data from both child and adult groups and compared the performance with the age-specific models.

Next, we sought to determine if the model trained and validated on the adult samples, the adult model, could be used to predict child ADHD, and vice versa. We hypothesized that if the ADHD vs. control sMRI differences seen in children are also present in adult ADHD brains, then the base models for each age group should be able to predict ADHD in the other age group. To create the largest test sets possible, we tested the child model on all the adult samples, and the adult model on all the child samples.

### Model ev**a**luation

The sigmoid function in the output layer of the neural network generates a continuous score that assesses the probability for each individual to be classified as ADHD. We name this continuous output the brain risk score. Using the brain risk scores, we calculated Cohen’s *d* effect sizes for child and adult test sets. We computed ROC curves and used the area under the ROC curve (AUC) as our primary measure of accuracy. The AUC and its confidence intervals were calculated in Stata 15 using the empirical method and compared with nonparametric approach by DeLong et al.^47^. We also computed precision-recall (PR) curves and reported the area under the PR curves, as well as the Brier loss for the final models as measures of accuracy and goodness of fit.

## Results

The screening results (Supplementary Fig. 1) showed that principal factors as input features greatly improved the classifiers’ performance compared with original MRI features, as demonstrated by higher validation AUCs achieved in many models. Using principal factors, MLP outperformed all other models and was chosen as the base model and used in the following main analysis after additional fine-tuning the hyperparameters. The final MLP models’ hyperparameters were listed in Supplementary Table 2.

**Fig. 1:**
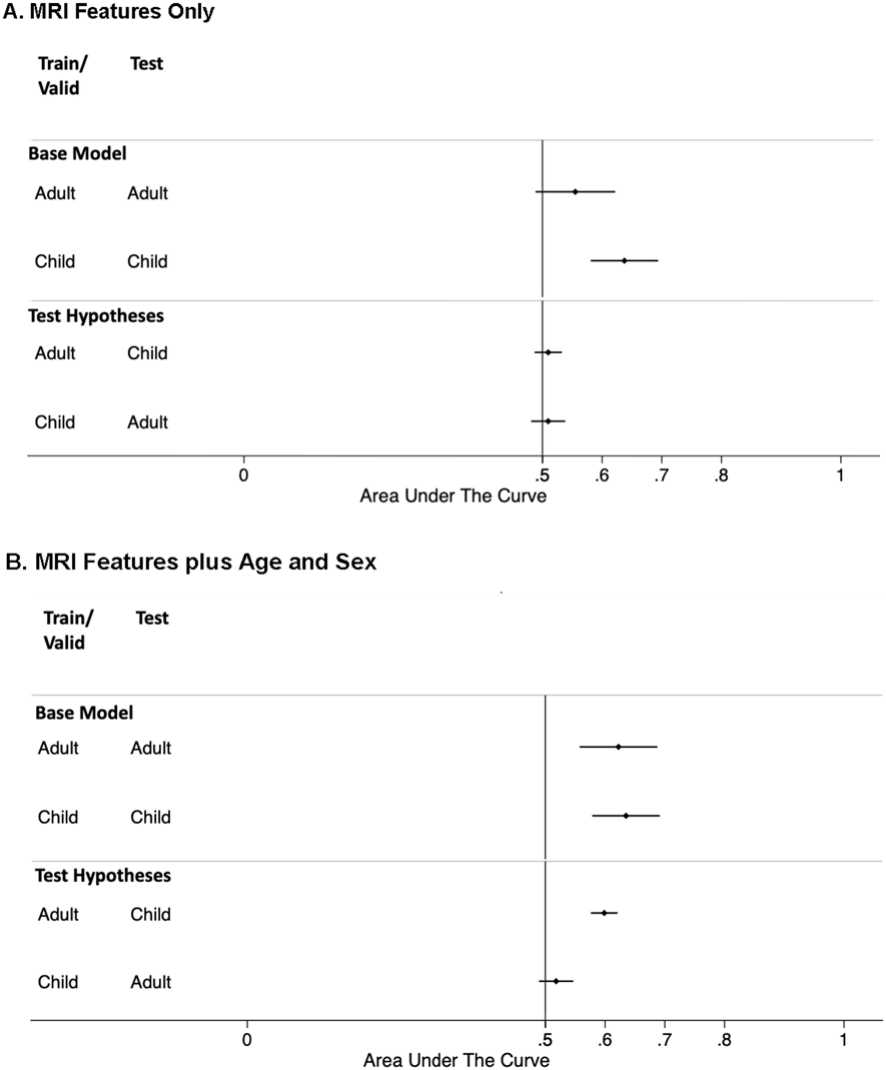
Area under the receiver operating characteristic curve for the test results. Area under the receiver operating characteristic curve (AUC) accuracy statistics for the held-out test results were plotted (as dots) with their 95% confidence intervals (as horizontal lines). The vertical line at an AUC of 0.5 indicates a chance level of diagnostic accuracy. If the 95%CI does not overlap with the 0.5 vertical line, it indicates significant predictive accuracy. **A** AUC comparison of the models using only MRI features. **A** AUC comparison of the models using MRI features plus age and sex. In both **A** and **B**, the Top portion shows the base models, where models were trained and validated in child or adult samples and tested on their corresponding age groups; Bottom portion tests the hypotheses that if model trained/validated on child samples can also predict adult ADHD and vice versa. Note that test sample consists of combined training, validation, and test sets from the other age group because they are not used in the model optimization and training.

Figure 1A (top portion) shows the test set AUCs (as dots) and their 95% confidence intervals (as horizontal lines) for the base models using only MRI factors. The model trained and validated on child data predicted child ADHD with a significant AUC 0.64 (95%CI 0.58–0.69). In contrast, the model trained and validated on adult data was not significant AUC (0.56, 95%CI 0.49–0.62, *p* = 0.057). ROC curves for the two base models are in Supplementary Fig. 2A. The difference between the two base models’ AUCs was not significant (X^2^_(1)_ = 3.4, *p* = 0.065). The areas under the precision-recall curve (AUPRC) were higher for the adult model (AUPRC = 0.74) than the child model (AUPRC = 0.68). Using the model predicted brain risk scores, we calculated the Cohen’s *d* effect sizes in the test set to be 0.47 for child samples (95%CI: 0.27–0.68) and 0.15 (−0.08–0.39) for the adult samples.

After adding age and sex as predictors, the adult model (Fig. 1B, top) increased the AUC to 0.62 (95%CI 0.56–0.69, *p* = 0.002). Although prediction AUC was now significant, the increase from the base model without age and sex was not statistically significant (X^2^_(1)_ = 2.01, *p* = 0.15). The AUPRC for the adult model also slightly increased to 0.79. Adding age and sex as predictors to the child model did not affect either the AUC, nor the AUPRC. ROC curves of two models are plotted in Supplementary Fig. 2B. The Cohen’s *d* effect sizes in the test set were 0.48 for children (95%CI: 0.27–0.69) and 0.39 (0.15–0.63) for adults. All above models had similarly small Brier scores (0.25).

The combined model with MRI features produced an overall test AUC of 0.60 (95%CI 0.55–0.64). The test AUC was 0.64 (95%CI 0.58–0.69) on the child subset and 0.54 (95%CI 0.47–0.60) on the adult subset, comparable to those from the age-specific models. Similarly, the combined model with MRI, age, and sex features produced an overall AUC of 0.63 (95%CI 0.59–0.67). The subset test AUC was 0.65 (95%CI 0.60–0.71) on the child subset and 0.56 (95%CI 0.49–0.63) on the adult subset, also statistically comparable to those of the age-specific models.

Because the training samples had been balanced for age and sex, these variables are not predictive of ADHD for either the child or adult test sets. To verify this, linear regression using only age and sex and their interactions to predict ADHD in the child and adult samples resulted in non-significant AUCs (child AUC 0.51, 95%CI: 0.45–0.57; adult AUC 0.46, 95%CI: 0.39–0.53).

### Tests of hypotheses

For models using only MRI features, neither the adult nor child models were successful at predicting ADHD in the other age group (Fig. 1A, bottom). However, the adult model that used both MRI features and age and sex was able to predict the child samples significantly (AUC = 0.60, 95%CI: 0.58–0.62, Fig. 1B bottom). The Cohen’s *d* effect size for children, based on the adult model predictions, was 0.17 (95%CI: 0.10–0.24), smaller than those predicted by their age-corresponding models. The child model that used both MRI features and age and sex did not significantly predict ADHD when applied to the adult samples (AUC = 0.53, 95%CI: 0.49, 0.56, Fig. 1B bottom). ROC curves of both models tested on the different age groups are plotted in Supplementary Fig. 2C.

## Discussion

Consistent with previous ENIGMA ADHD findings^31,32^, we found that the ability of sMRI data to discriminate people with and without ADHD is much stronger for children than adults, which is consistent with a broader literature showing that ADHD-associated structural brain differences diminish with age^19,34–37^. While the ENIGMA ADHD study did not find any significant differences between ADHD and control subjects for adults, our adult model did achieve a significant AUC 0.62 (95%CI 0.56–0.69) and a high area under the PR curve (AUPRC = 0.79). Consistent with the ENIGMA findings, our model-predicted brain risk scores had a larger effect size for the children than adults in both the models using MRI features and those with age and sex added. Notably, our effect sizes were two times greater than the largest of those individual regions reported in prior ENIGMA ADHD studies for both children (Cohen’s *d* = −0.21) and adults (Cohen’s *d* = −0.16)^31,32^.

Only a handful of prior ML studies attempted to classify ADHD from controls using only sMRI data. Most used resting-state functional MRI (rs-fMRI), or rs-fMRI in combination with another MRI modality, sometimes including cognitive measurements such as IQ. Many prior studies reported model performance on a cross-validation dataset without using an independent test set. We and many others have warned about the risk of data leakage and model overfitting when using only cross-validation without an independent test set^26,48–51^. Among those that reported independent test results, classification accuracies varied from 37 to 93%, with an average of 68% (ref. ^26^). Notably, it is difficult to directly compare the accuracy scores with our AUC scores since many of these studies used imbalanced datasets. Nevertheless, one study, among those, reported classifiers built with only sMRI features. In that study, Yoo and colleagues examined various combinations of fMRI, sMRI features, and genetic data from a balanced cohort of 94 children and adolescents. The unimodal sMRI classifier, using the cortical thickness and volumes, achieved an accuracy of 69.4% and AUC 0.65 in a small independent test set (18 ADHD and 18 typically developing children)^52^. Although the AUC is comparable to our child model, it is not clear how well this model would generalize to other samples given the extremely small sample sizes in both training and test sets. Nevertheless, the authors reported a better AUC (0.70) with a multimodal classifier built with features from both diffusion tensor imaging and sMRI data^52^.

Although our results from the child and adult base models show that sMRI data are not sufficiently predictive to be useful in clinical practice, they provide crucial pieces of evidence that will be useful in future attempts at predictive modeling. We are the first to confirm in the largest possible adult ADHD MRI sample available, that adults with ADHD differ significantly from adults without ADHD on sMRI features. Only a few prior studies attempted to classify adult ADHD from controls, but all used extremely small dataset (<100 (refs. ^28–30^)). Although these studies reported higher accuracies (74%–80%), all were based on cross-validation results and none reported prediction performance on independent test sets. The improvements we found by adding age and sex to the adult model indicate that these demographic variables must moderate the predictive ability of sMRI features. These demographics moderate the sMRI effects because our regression models show that the demographic variables on their own have no predictive utility (which was fixed in advance by balancing the case and control training samples by age and sex). It is possible that there are different age subgroups within the adult dataset that demonstrate different patterns of MRI features. For example, many regions of the brain, including prefrontal cortex, do not fully mature until early adulthood, around age 25 (ref. ^53^). Perhaps the age group “adults” should not include developing brains prior to age 25. However, we cannot assess for such age effects due to the sample sizes of more refined age groups. Future work should recruit more MRI data particularly for under-represented adolescent and older adult age groups. We have also shown that ML methods dramatically increase the ADHD vs. Control effect size compared with the prior univariate ENIGMA analyses.

The results from our hypothesis testing provide further information that is useful in understanding the continuity of child and adult ADHD. Consistent with our hypothesis, the adult model, trained only on adult samples, significantly predicted ADHD in the child samples. This suggests that the adult model learned combinations of structural features relevant for discriminating the sMRI scans from children with and without ADHD. This implies that some of ADHD’s sMRI differences that are relevant for persistent cases are also relevant in childhood (only some of which will be persistent into adulthood). This conclusion must, however, be considered equivocal because the child model did not successfully predict ADHD in the adult samples. To resolve this issue, future studies will need to find a way to better discriminate sMRI features associated with the onset of ADHD and those associated with the persistence of ADHD.

Our work should be interpreted in the context of several limitations. First, because we combined data across many sites, we inherit all the limitations of the original studies. Heterogeneity of methods across studies may have added noise to the combined dataset that made it difficult to discriminate the data from people with and without ADHD. Second, we only used structural imaging data. Incorporating other imaging modalities might provide clearer results and conclusions. Third, we used pre-defined structures from ENIGMA standard image processing pipeline as features. It is possible that other methods such as one using 3D images as input features, in a convolutional neural network, would uncover useful features leading to increased classification accuracy. However, the 3D images are not available. Finally, our use of neural networks makes it difficult to clarify the importance of each brain region in the model’s algorithm.

Despite these limitations, we have shown that a neural network approach is able to detect case-control sMIR differences in adults with ADHD that could not be detected with standard analyses. We have also provided some evidence for the continuity of sMRI findings from childhood into adulthood.

## Supplementary information

Supplemental Materials

## Data Availability

The machine learning codes were freely accessible from the GitHub repository (https://github.com/ylzhang29/ADHD_MLP) for research purposes.

## Supplementary information

**Supplementary information** The online version contains supplementary material available at 10.1038/s41398-021-01201-4.
