# Supplementary material for: Evidence for similar structural brain anomalies in youth and adult attention-deficit/hyperactivity disorder: a machine learning analysis
Source: Transl Psychiatry. 2021 Feb 1;11:82. doi: 10.1038/s41398-021-01201-4 (PMC7851168; doi:10.1038/s41398-021-01201-4)
Supplement: Supplementary file 1 — Supplemental Materials [file 41398_2021_1201_MOESM1_ESM.docx]

**Supplementary Figure 1. Comparing input features and different machine learning models.**

Grid search results for nine different ML models are shown. Each dot represents one model with a unique set of hyperparameters. **Left** figure shows models using the original MRI features and the **Right** figure shows models using the 46 principal factors. For all the models, training AUCs were plotted on y axis and validation AUCs were on the x axis. The red arrow points to the model using MLP that had a high validation AUC and low training AUC.

The details of each model and their hyperparameters spaces are listed: 1) Decision Tree Classifier: criterion: ["gini", "entropy"]; splitter: ["best", "random"]; max_depth: [1,3,5,10,15, 20, 30,50]; max_features: [.1, .2, .3, .4, .5]. 2) Random Forests: max_features: [.1, .2, .3, .4, .5 ]; n_estimators: [10, 20, 50, 100, 500, 1000, 2000]; max_depth : [1,3,5,10, 15, 20]. 3) Support Vector Machine: C: [.01, 1, 10, 100, 1000, 10000]; gamma: [.01, .1, 1, 10, 100, 1000, 10000]. 4) Ridge Classifer: alpha: [0.001, 0.01, .1, .2, .3, .4, .5, .6, .7, .8, .9, 1.0]; tol: [0.00001,0.0001, 0.0005, 0.001, 0.005,.01]. 5) k-Nearest Neighbors: n_neighbors: [5,10,30,40,50,100,500]; leaf_size: [5,10,15,20,30,50,100]; p: [1, 2]. 6) Lasso: alpha: [0, 0.001, 0.01, .1, .3, .5,.7, 1.0]; tol: [0.00001,0.0001, 0.0005, 0.001, 0.005,.01]. 7) Logistic Regression: C: [0.00001,0.0001, 0.001, 0.01, .1,.5, 1.0]; tol: [0.0001, 0.0005, 0.001, 0.005]; solver:["liblinear", "newton-cg","lbfgs", "sag"]. 8) Elastic Net: alpha': [0.001, 0.01, .1, .3, .5,.7, 1.0]; l1_ratio: [.1, .3, .5,.7, 1.0]; tol: [0.0001, 0.0005, 0.001, 0.005]. 9) MLP: Numbers of hidden layers: [1, 2, 3, 5, 10]; hidden_layer_sizes: [10, 50, 100, 200, 500]; alpha/L2 regularizer: [.00001, 0.0001, 0.001]; learning_rate_init: [0.0001, 0.0005, 0.001, 0.01].

**
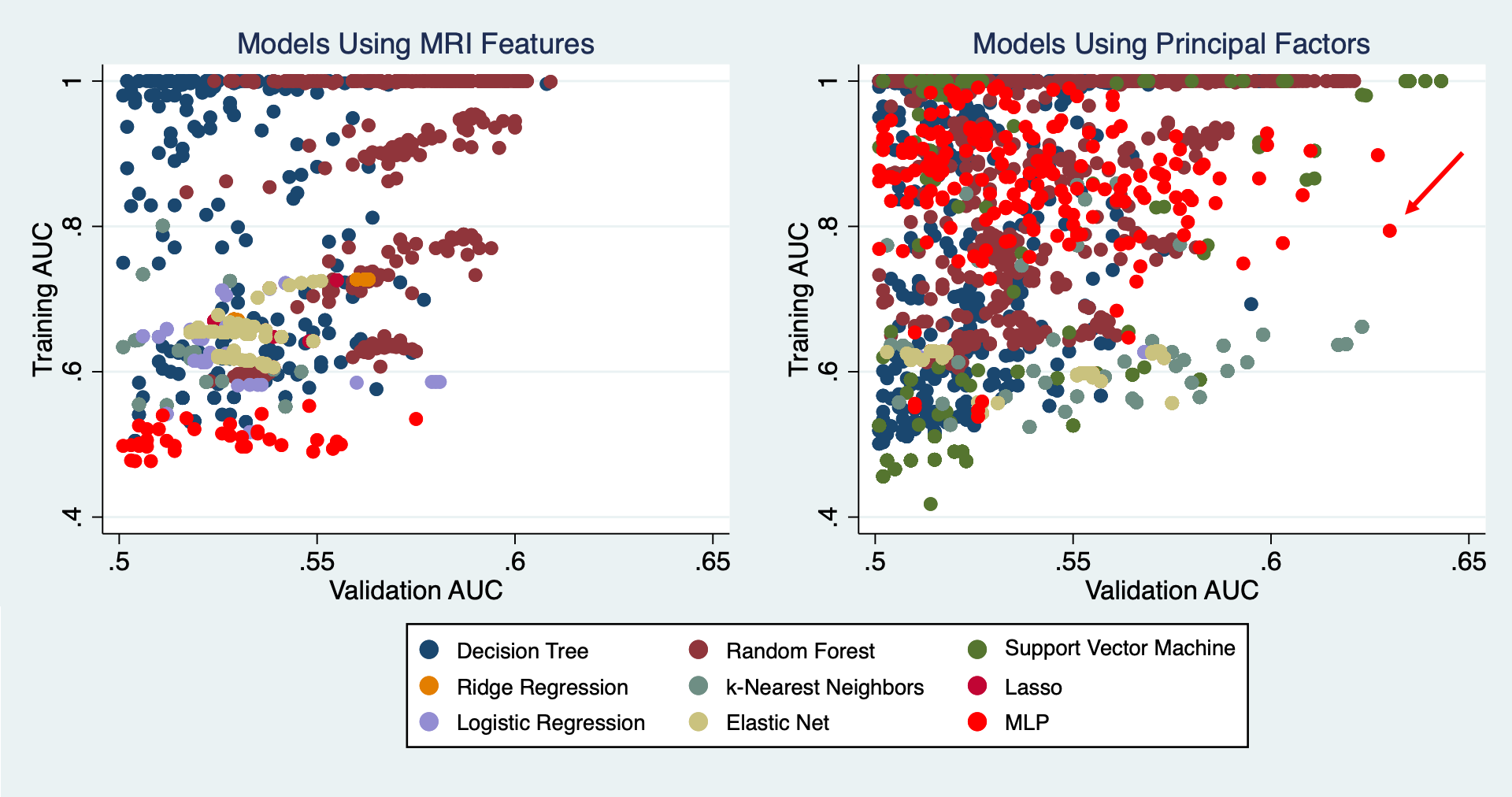
Supplementary Figure 2.** Receiver operating characteristic (ROC) curves of the base models and those tested on the different age groups.

**A.** Base model using MRI features only


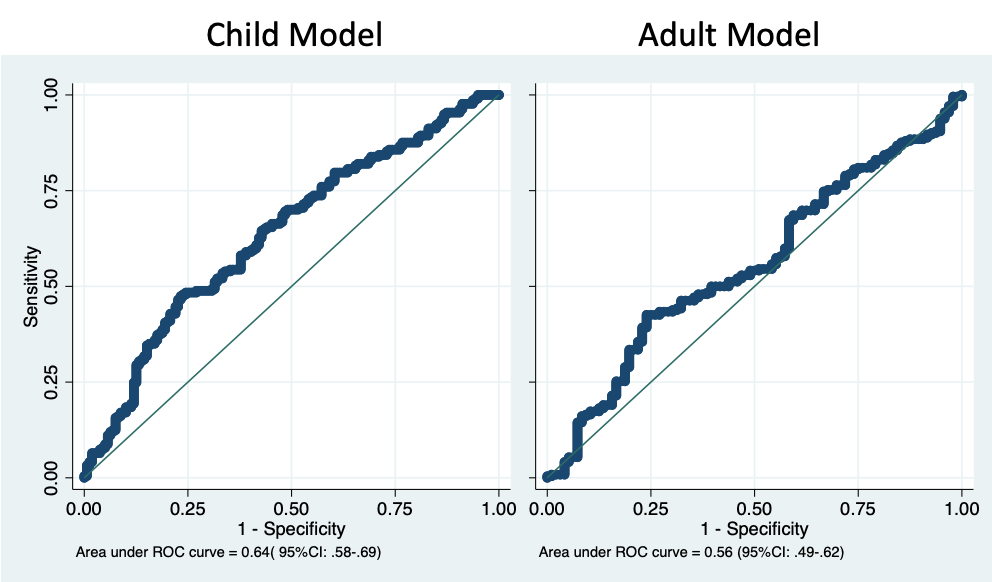


**B.** Model using MRI features and age, sex
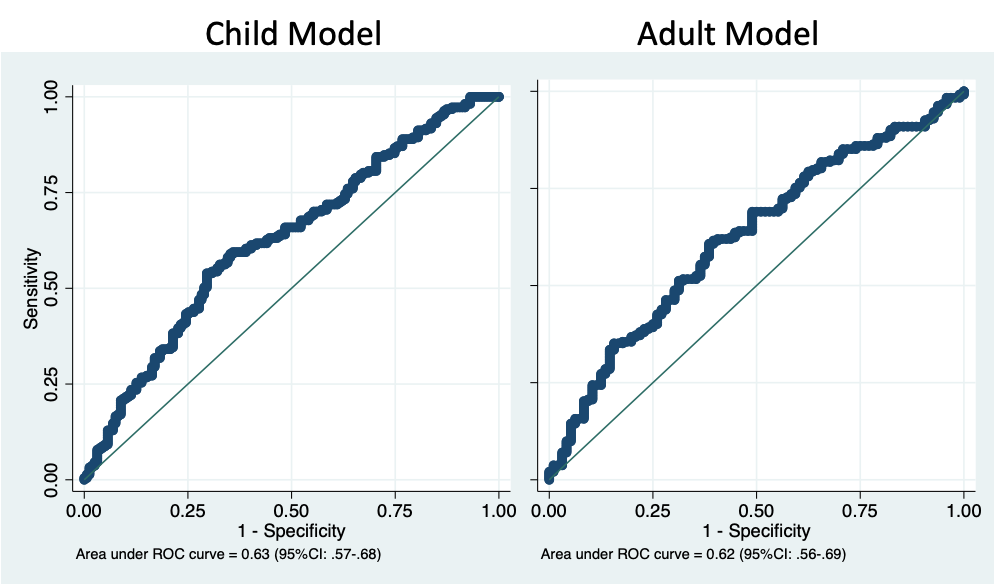


**C.** Tests of hypotheses: Models (using MRI features and age, sex) were tested on different age groups.


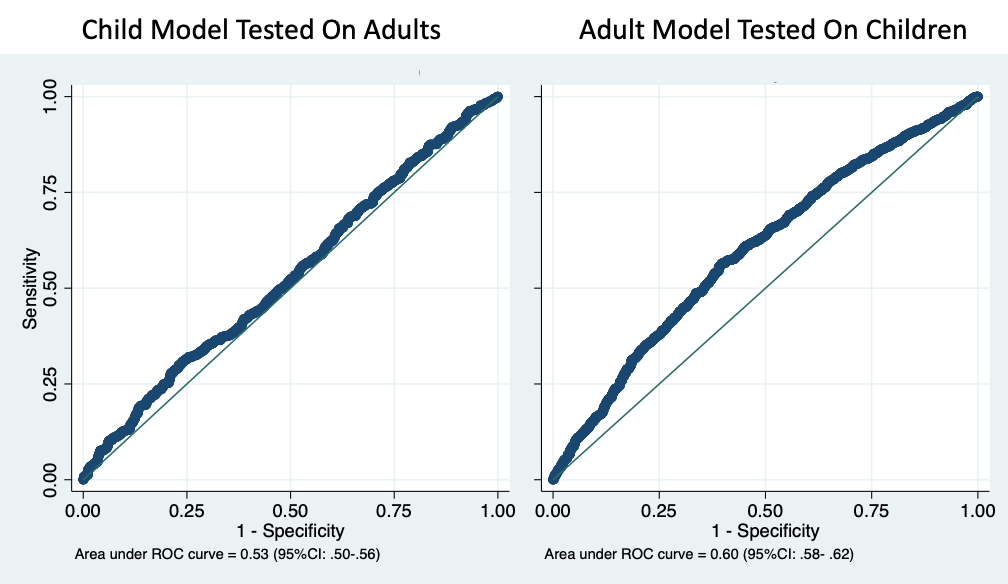


| **Supplementary Table 1. Total sample and train/validation/test splits from each site** | | | | | |
| --- | --- | --- | --- | --- | --- |
| **Sites** | **Training** | **Validation** | **Test** | **Excluded** | **Total** |
| ACPU | 46 | 11 | 9 | 1 | 67 |
| ADHD_WUE | 75 | 18 | 14 | 0 | 107 |
| ADHD-DUB1 | 56 | 14 | 10 | 0 | 80 |
| ADHD-DUB2 | 0 | 0 | 0 | 20 | 20 |
| ADHD-Mattos | 0 | 0 | 0 | 31 | 31 |
| ADHD200_KKI | 61 | 14 | 10 | 0 | 85 |
| ADHD200_NYU | 158 | 36 | 31 | 3 | 228 |
| ADHD200_OHSU | 61 | 16 | 12 | 0 | 89 |
| ADHD200_Peking | 139 | 31 | 27 | 0 | 197 |
| ADHDAachen | 100 | 24 | 20 | 1 | 145 |
| ADHD_Rubia | 45 | 11 | 9 | 6 | 71 |
| ADHD_Russia | 0 | 0 | 0 | 10 | 10 |
| Barcelona | 51 | 12 | 10 | 0 | 73 |
| Bergen_SVG | 35 | 10 | 6 | 0 | 51 |
| Bergen_adultADHD | 55 | 15 | 11 | 0 | 81 |
| CAPS_UZH | 41 | 9 | 5 | 0 | 55 |
| DAT_london | 38 | 11 | 7 | 0 | 56 |
| Dundee | 32 | 8 | 4 | 1 | 45 |
| EPOD | 0 | 0 | 0 | 92 | 92 |
| Hartford_Olin | 125 | 31 | 25 | 0 | 181 |
| IMpACT_NL | 188 | 42 | 38 | 0 | 268 |
| MGH_ADHD | 100 | 24 | 20 | 0 | 144 |
| MTA | 91 | 21 | 17 | 0 | 129 |
| NICAP | 102 | 24 | 20 | 0 | 146 |
| NIH | 282 | 63 | 59 | 9 | 413 |
| NYU_ADHD | 56 | 14 | 10 | 0 | 80 |
| NeuroImage_ADAM | 118 | 29 | 21 | 0 | 168 |
| NeuroImage_NIJM | 120 | 30 | 22 | 0 | 172 |
| OHSU2018 | 161 | 36 | 32 | 0 | 229 |
| SAOPAULO | 92 | 22 | 18 | 1 | 133 |
| Sussex | 40 | 11 | 7 | 0 | 58 |
| Tuebingen | 0 | 0 | 0 | 28 | 28 |
| UAB-ADHD | 138 | 34 | 26 | 0 | 198 |
| UCHZ | 54 | 16 | 8 | 0 | 78 |
| ZI-CAPS | 24 | 7 | 3 | 0 | 34 |
| Total | 2,684 | 644 | 511 | 203 | 4,042 |

**Supplementary Table 2** Sample subsets from each site.

| Training and Validation Samples | Feature | Hyperparameters | | | | | | |
| --- | --- | --- | --- | --- | --- | --- | --- | --- |
|  |  | Activation Function | Optimizer | Numbers of layers | Numbers of Units | Batch Size | dropout | Learning Rate |
| Combined | 46 Factors | relu | Adagrad | 1 | 267 | 52 | 0.357199 | 0.0018 |
| Adult | 46 Factors | relu | Adadelta | 1 | 35 | 104 | 0.177828 | 0.00097 |
| Child | 46 Factors | relu | Adagrad | 1 | 260 | 124 | 0.868807 | 0.0032 |
| Combined | 46 Factors + Age, Sex | relu | RMSprop | 1 | 243 | 124 | 0.787445 | 0.0055 |
| Adult | 46 Factors + Age, Sex | selu | Adadelta | 1 | 107 | 188 | 0.495459 | 0.0074 |
| Child | 46 Factors + Age, Sex | relu | Adagrad | 1 | 432 | 12 | 0.216935 | 0.0022 |
